# Validating muscle mass cutoffs of four international sarcopenia‐working groups in Japanese people using DXA and BIA

**DOI:** 10.1002/jcsm.12732

**Published:** 2021-06-07

**Authors:** Yosuke Yamada, Minoru Yamada, Tsukasa Yoshida, Motohiko Miyachi, Hidenori Arai

**Affiliations:** ^1^ Department of Physical Activity Research National Institutes of Biomedical Innovation, Health and Nutrition Tokyo Japan; ^2^ Faculty of Human Sciences University of Tsukuba Tsukuba Japan; ^3^ National Center for Geriatrics and Gerontology Aichi Japan

**Keywords:** Biomarkers, Sarcopenia, Dual‐energy X‐ray absorptiometry, Bioelectrical impedance analysis, Diagnosis, Muscle diseases

## Abstract

**Background:**

The Asian Working Group for Sarcopenia (AWGS) 2019 recommended the use of dual‐energy X‐ray absorptiometry (DXA) or bioelectrical impedance analysis (BIA) to assess appendicular lean mass (ALM). AWGS, European Working Group on Sarcopenia in Older People 2 (EWGSOP2), Foundation for the National Institutes of Health Sarcopenia Project (FNIH), and International Working Group on Sarcopenia (IWGS) reported different cutoff values for sarcopenia. We aimed to validate these cutoff values in a Japanese population using DXA and two different devices of segmental multi‐frequency BIA (MF‐BIA).

**Methods:**

We examined the data of Japanese individuals aged 18–86 years using the DXA (*n* = 756) and two 8‐electrode MF‐BIA devices (InBody and TANITA MC) (*n* = 1884). To validate these cutoff values, we used a population aged 18–40 years, and calculated the 95% confidence intervals (CIs) of [mean−2SD].

**Results:**

In DXA, the 95%CIs of [mean−2SD] for ALM/Ht^2^ were 5.2–5.8 and 6.6–7.3 kg/m^2^ in women and men, respectively. The AWGS (<5.4 in women and <7.0 in men), and IWGS (≤5.67 in women and ≤7.23 in men) cutoffs were acceptable. Regarding TANITA MC, the 95%CIs of [mean−2SD] for ALM/Ht^2^ were 5.6–6.0 and 6.9–7.4 kg/m^2^ in women and men, respectively. The AWGS (<5.7 in women and <7.0 in men), EWGSOP2 (<6.0 in women and <7.0 in men), and IWGS cutoffs were acceptable. Regarding InBody, the 95%CIs of [mean−2SD] for ALM/Ht^2^ were 4.8–5.2 and 6.4–6.8 kg/m^2^ in young women and men, respectively. All cutoff values were too high compared to those measured by InBody. InBody and TANITA MC were highly correlated (*P* < 0.001), but the values by InBody were significantly lower than those by TANITA MC or DXA. Using Yamada's equation for InBody raw data, the AWGS, EWGSOP2, or IWGS cutoffs were acceptable. The BMI‐adjusted muscle mass cutoff values were <0.60 and <0.82 m^2^ in women and men, respectively. We also obtained the 20th percentile in older adult population (ALM/Ht^2^, <6.2 in women and <7.5 in men for TANITA MC; <5.4 in women and <7.0 in men for InBody).

**Conclusions:**

The AWGS and IWGS cutoffs were valid for DXA, and the AWGS, IWGS, and EWGSOP2 cutoffs were valid for TANITA MC in Japanese population. Because the prevalence of sarcopenia is too low particularly in women when using those criteria, the 20th percentile might be a good alternative criteria. If the ALM original InBody values are used, the cutoffs should be <5.0 kg/m^2^ in women and <6.6 kg/m^2^ in men.

## Introduction

Assessment of skeletal muscle mass and its cutoff values is a fundamental issue for sarcopenia diagnosis.^1^, ^2^, ^3^, ^4^, ^5^, ^6^, ^7^, ^8^ These cutoff values are considered method‐ and device‐dependent and also ethnicity‐dependent.^9^ The Asian Working Group for Sarcopenia (AWGS) 2019 recommends the use of dual‐energy X‐ray absorptiometry (DXA) or multi‐frequency bioelectrical impedance analysis (MF‐BIA) (both are squared‐height‐adjusted) for muscle‐mass measurement (kg/m^2^) to diagnose sarcopenia in clinical settings.^7^ The AWGS 2019 cutoffs for low muscle mass (LMM) to diagnose sarcopenia are as follows: <7.0 kg/m^2^ in men and <5.4 kg/m^2^ in women using DXA; and <7.0 kg/m^2^ in men and <5.7 kg/m^2^ in women using BIA.^7^ However, particularly, MF‐BIA is a secondary indirect method to assess skeletal muscle mass, and variations in estimation methods between companies may exist. When systematic differences are observed between devices, the prevalence of sarcopenia might be affected by these differences. Moreover, the AWGS,^7^ European Working Group on Sarcopenia in Older People 2 (EWGSOP2),^8^ Foundation for the National Institutes of Health Sarcopenia Project (FNIH),^4^ and International Working Group on Sarcopenia (IWGS)^5^ have reported different cutoff values for sarcopenia. Therefore, there is a requirement for validating those cutoff values simultaneously.

In addition, The FNIH Sarcopenia Project indicated that body mass index (BMI)‐adjusted muscle mass may be superior to unadjusted muscle mass in predicting functional outcomes and disability in older adults.^3^, ^4^ The AWGS 2019 consensus^7^ stated that the FNIH criteria (i.e., <0.789 kg/BMI in men and <0.512 kg/BMI in women) may be appropriate cutoff values (DXA‐measured muscle mass only).^3^, ^4^ However, the mean values and distribution of BMI differ between the United States (US) (or European) and Asian populations.^10^ In general, the East Asian population has lower mean BMI and body fat percentage than the corresponding US population, and consequently the cutoff values should be different among various populations.^10^ Adequate cutoff values for BMI‐adjusted muscle mass in Asian populations are also required.

To validate the cutoff values, it was necessary to consider that the means and standard deviations (SDs) of the sample were not the same as the means and SDs of the population; both means and SDs have confidence intervals (CIs). We had to consider the 95%CI of the means and 95% CI of SDs when the value of [mean‐2SD] was calculated.^11^ No previous studies have shown the range of the value of [mean‐2SD]. Here, we calculated the 95%CIs of means and SDs and compared the results with the current consensus. The aims of the current study were as follows: (i) to validate the AWGS, EWGSOP2, FNIH, and IWGS cutoff values in the Japanese population; (ii) establish cutoff values for BMI‐adjusted muscle mass using one DXA device and two different devices of segmental MF‐BIA; and (iii) obtain the 20th percentile in older adult population to establish an alternative criteria of LMM.

## Methods

The current study comprises two resources: the DXA and MF‐BIA studies. The DXA study was previously reported by Yamada et al. (2017)^12^ and included 756 healthy Japanese individuals (437 women and 319 men) aged 18–86 years. The DXA study protocol was approved by the Ethics Committee of TANITA Co. IRB (approval numbers: #004, 005, 010, and 012). The MF‐BIA study was previously reported by Yamada et al. (2016),^13^ in which 1884 healthy Japanese aged 18–89 years participated. Among these participants, 16 (0.8%) had incorrect entry of sex or height into devices. Therefore, a total of 1868 participants' data were analysed in this study (1,163 women and 705 men). The MF‐BIA study protocol was approved by the Ethics Committee of the Faculty of Human Sciences, University of Tsukuba (approval number: 26–28). We excluded patients with cancer, stroke, congestive heart failure, chronic obstructive pulmonary disease, and arthritis. All participants provided written informed consent after reviewing the purpose, methods, and significance of the study. Thereafter, we measured the height (Ht) and weight of the participants. The minimum Ht and weight graduation were 0.1 cm and 0.1 kg, respectively. The inclusion criteria were: (i) reported ability to walk >10 m with or without a cane; (ii) ability to provide informed consent with no signs of dementia; and (iii) no history of joint arthroplasty or current use of an artificial pacemaker.

### DXA

A Lunar DPX‐L (GE Healthcare, Madison, WI, USA) densitometer was used, and analyses were performed using the software version 1.35 (Lunar DPX‐L ver1.35, GE Healthcare) to estimate appendicular lean mass (ALM). Routine densitometry quality assurance procedures were conducted using a standard phantom once per week and calibrated before the test. Moreover, no instrument drift or shift was detected during the measurement period.^12^

### Mf‐BIA

Two standing‐posture 8‐electrode MF‐BIA devices: MC‐980A (TANITA, Tokyo, Japan) and InBody 770 (InBody Corp., Seoul, Korea), were used to measure bioelectrical impedance.^13^ The order, in which measurements were performed, was randomized. Both systems used electrical current at different frequencies (1, 5, 50, 250, 500, and 1,000 kHz) to estimate the amounts of extracellular and intracellular water in the body. The study participants stood on two metallic electrodes and held metallic grip electrodes.

ALM using TANITA MC‐980A was calculated using the following, previously developed equation by Yamada et al.^12^:

$$\;\mathrm{ALM}=\left(0.6947\times \left({\mathrm{Ht}}^{2}/Z_{50}\right)\right)+\left(-55.24\times \left(Z_{250}/Z_{5}\right)\right)+\left(-10,940\times \left(1/Z_{50}\right)\right)+51.33\;\text{for}\;\mathrm{men}.$$

$$\;\mathrm{ALM}=\left(0.6144\times \left({\mathrm{Ht}}^{2}/Z_{50}\right)\right)+\left(-36.61\times \left(Z_{250}/Z_{5}\right)\right)+\left(-9332\times \left(1/Z_{50}\right)\right)+37.91\;\text{for}\;\mathrm{women}.$$

The theoretical reason for each variable is as follows: Most BIA devices and equations use an impedance (Ht^2^/Z_50_) or resistance index of 50 kHz (Ht^2^/R_50_) calculated by Ht and Z_50_, or resistance at 50 kHz (R_50_) as a predicting variable for ALM.^14^, ^15^ In the human body, reactance is <10% of resistance, and the correlation between impedance and resistance is >0.99; thus, Ht^2^/Z_50_ and Ht^2^/R_50_ are interchangeable. Expansion of extracellular water (ECW) relative to intracellular water (ICW) or total body water (TBW) is observed with aging, and may mask actual age‐related decreases in the muscle cell mass. The Z at low‐frequency (≤50 kHz, for example, Z_5_ or Z_50_) currents mainly reflects the ECW.^16^ In contrast, the Z at a high frequency (≥250 kHz) reflects the TBW. Thus, the impedance ratio of Z_250_ to Z_5_ (Z_250_/Z_5_) is an index of ECW/TBW, and Z_250_/Z_5_ is an independent variable to estimate ALM. In addition, possibility of edema affects ALM estimation: when an individual has edema, particularly peripheral edema, the BIA overestimates actual ALM. The index of 1/Z_5_ or 1/Z_50_ could potentially be applied as an adjusting variable for this situation. As body composition, fat, and muscle distribution are different between men and women, the estimating equations for men and women were established separately.

ALM using InBody 770 was measured by the original formula inherent to the device (equations not disclosed), and Yamada's equation as follows:

$$\;\mathrm{ALM}=\left(0.6947\times \left({\mathrm{Ht}}^{2}/Z_{50}\right)\right)+\left(-55.24\times \left(Z_{250}/Z_{5}\right)\right)+\left(-10,940\times \left(1/Z_{50}\right)\right)+51.33\;\text{for}\;\mathrm{men}.$$

$$\;\mathrm{ALM}=\left(0.6144\times \left({\mathrm{Ht}}^{2}/Z_{50}\right)\right)+\left(-36.61\times \left(Z_{250}/Z_{5}\right)\right)+\left(-9332\times \left(1/Z_{50}\right)\right)+37.91\;\text{for}\;\mathrm{women}.$$

A previous study showed that the reference value (SMI) for LMM in each sex was defined as a value of 2 SDs below the sex‐specific means of the study reference data of young adults aged 18–40 years.^17^ In the previous study, the study population included young adults (19,797 men and 18,302 women) aged 18–40 years to determine the reference values. The SMIs in young men and women aged 18–40 years were 8.11 ± 0.68 and 6.35 ± 0.64 kg/m^2^, respectively. Therefore, the reference values of LMM in Japanese men and women using InBody original values had been set at 6.75 and 5.07 kg/m^2^, respectively.^17^

### Statistical analysis

The results are presented as means ± SD, with maximum and minimum values. The participants were divided into three groups according to their age: 18–40 years (young adults), 41–64 years, and 65–89 years (older adults). As physical characteristics differed between sexes, statistical analyses were applied to men and women separately. To calculate the 95% CIs of [mean‐2SD], the values of the 95%CI of the mean and the SD at the 95%CI were obtained.^11^ Quadratic regression analyses were performed to examine the associations between age and other variables. We also obtained the 20th percentile of the ALM, ALM/Ht^2^, and ALM/BMI values of the older adult cohort. All analyses were performed using SPSS software (Version 22.0; IBM Corp., Armonk, NY, USA). For all analyses, values of *P* < 0.05 were used to indicate statistical significance.

## Results

Table 1 shows the Ht, weight, BMI, ALM, ALM/Ht^2^, 95%CI of [mean‐2SD], and percentage of those under the AWGS cutoff value of LMM in female participants aged 18–40 years. The weights measured by the TANITA and InBody devices were similar. Ht, weight, and BMI were not significantly different between the BIA and DXA cohorts (*P* > 0.05). The ALM value estimated by TANITA MC (17.4 ± 2.0 kg) was not significantly different from that measured by DXA (18.0 ± 2.7 kg). However, the ALM value estimated by InBody 770 (15.8 ± 2.2 kg) was significantly lower than that measured by DXA (*P* < 0.001). No prevalence of LMM according to the AWGS cutoff values was found in TANITA MC and DXA. However, it was significantly higher in the InBody (21.4%) than in other cohorts in women aged 18–40 years. When Yamada's equation was applied to InBody raw impedance data, the ALM and ALM/Ht^2^ values were matched with those calculated by TANITA MC and DXA. The [mean–2SD] of ALM/Ht^2^ measured by the TANITA MC (95%CI, 5.6–6.0 kg/m^2^), InBody recalculated using Yamada's equation (5.6–6.0 kg/m^2^), and DXA (5.2–5.8 kg/m^2^) devices were not significantly different from the AWGS cutoff values for LMM (*P* > 0.05). However, The 95%CI of [mean–2SD] of ALM/Ht^2^ in the InBody original values (4.8–5.2 kg/m^2^) was significantly lower than the AWGS cutoff values, and The [mean‐2SD] of ALM/Ht^2^ in the InBody original values (5.0 kg/m^2^) was not significantly different from the previously reported cutoff value (5.07 kg/m^2^). The 95%CI of [mean‐2SD] for ALM/BMI based on the present female participants aged 18–40 years ranged from 0.55 to 0.64 in the TANITA MC, InBody recalculated using Yamada's equation, and DXA cohorts. Therefore, we set the cutoff value for ALM/BMI at <0.60m^2^. The cutoff value for ALM/BMI was <0.55 in the InBody original values.

**Table 1:** Physical characteristics, ALM, ALM/Ht^2^, and 95%CI of [mean‐2SD] in female participants aged 18–40 years

	TANITA MC	InBody (Original)	InBody (Yamada's equation)	DXA
	*n* = 154	n = 154	n = 154	*n* = 189
Age (years)	28.6 ± 6.3	28.6 ± 6.3	28.6 ± 6.3	27.9 ± 5.8
Height (cm)	159.2 ± 5.4	159.2 ± 5.4	159.2 ± 5.4	158.8 ± 5.3
Weight (kg)	53.2 ± 7.7	53.2 ± 7.7	53.2 ± 7.7	53.4 ± 7.5
BMI (kg/m^2^)	21.0 ± 2.9	21.0 ± 2.9	21.0 ± 2.9	21.2 ± 2.6
ALM (kg)	17.4 ± 2.0	15.8 ± 2.2***	17.4 ± 2	18.0 ± 2.7
ALM/Ht^2^ (kg/m^2^)	6.9 ± 0.5	6.2 ± 0.6***	6.9 ± 0.5	7.1 ± 0.8
ALM/BMI (m^2^)	0.84 ± 0.12	0.76 ± 0.11***	0.84 ± 0.12	
[mean‐2SD] of ALM/Ht^2^ (kg/m^2^)	5.85	5.01	5.83	5.49
95%CI of [mean‐2SD] for ALM/Ht^2^	5.6―6.0	4.8―5.2*	5.6―6.0	5.2―5.8
% of AWGS Low Muscle Mass	0.0%	21.4%†††	0.0%	0.0%
95%CI of [mean‐2SD] for ALM/BMI	0.55―0.64	0.50―0.58	0.55―0.64	0.55―0.64

BMI, body mass index; ALM, appendicular lean mass; Ht, height; CI, confidence interval; AWGS, Asian Working Group for Sarcopenia.

*** *P* < 0.001,

* *P* < 0.05, significantly lower than other values.

††† *P* < 0.001, significantly higher than other values.

Table 2 shows the Ht, weight, BMI, ALM, ALM/Ht^2^, 95%CI of [mean‐2SD], and percentage of those under the AWGS cutoffs of LMM in male participants aged 18–40 years. The measured weight was similar between the TANITA and InBody devices. Ht, weight, and BMI were not significantly different between the BIA and DXA cohorts (*P* > 0.05). The ALM estimated by TANITA MC (26.1 ± 3.5 kg) was not significantly different from that measured by DXA (25.5 ± 3.2 kg). However, the ALM estimated by InBody 770 (23.8 ± 3.1 kg) was significantly lower than that measured by DXA (*P* < 0.001). The prevalence of LMM according to the AWGS cutoffs was significantly higher after using the InBody (21.4%) than the TANITA MC device. When Yamada's equation was applied to the InBody raw impedance data, the ALM and ALM/Ht^2^ values matched with those obtained using the TANITA MC and DXA. The [mean‐2SD] of ALM/Ht^2^ in the TANITA MC (95%CI, 6.9–7.4 kg/m^2^), InBody recalculated using Yamada's equation (6.9–7.4 kg/m^2^), and DXA (6.6–7.3 kg/m^2^) were not significantly different from the AWGS cutoff values for LMM (*P* > 0.05). However, the 95%CI of [mean–2SD] of ALM/Ht^2^ in InBody original values (6.4–6.8 kg/m^2^) was significantly lower than the AWGS cutoff, and the [mean‐2SD] of ALM/Ht^2^ of InBody original values (6.6 kg/m^2^) was not significantly different from the previously reported cutoff value (6.75 kg/m^2^). The 95%CIs of [mean‐2SD] for ALM/BMI based on the present male participants aged 18–40 years were 0.76–0.87, 0.76–0.86, and 0.77–0.89 m^2^ in TANITA MC, InBody recalculated using Yamada's equation, and DXA cohorts, respectively; thus, we set the ALM/BMI cutoff value at <0.82 m^2^. The cutoff value for ALM/BMI was <0.75 m^2^ in InBody original values.

**Table 2:** Physical characteristics, ALM, ALM/Ht^2^, and 95%CI of [mean‐2SD] in male participants aged 18–40 years

	TANITA MC	InBody (Original)	InBody (Yamada's equation)	DXA
	n = 212	*n* = 212	n = 212	*n* = 139
Age (years)	27.5 ± 6.3	27.5 ± 6.3	27.5 ± 6.3	29.2 ± 5.4
Height (cm)	171.9 ± 6.4	171.9 ± 6.4	171.9 ± 6.4	171.3 ± 5.4
Weight (kg)	68.1 ± 10.9	68.1 ± 10.9	68.1 ± 10.9	67.3 ± 9.7
BMI (kg/m^2^)	23.0 ± 3.3	23.0 ± 3.3	23.0 ± 3.3	22.9 ± 3.1
ALM (kg)	26.1 ± 3.5	23.8 ± 3.1***	26.0 ± 3.5	25.5 ± 3.2
ALM/Ht^2^ (kg/m^2^)	8.8 ± 0.8	8.0 ± 0.7***	8.8 ± 0.8	8.7 ± 0.9
ALM/BMI (m^2^)	1.15 ± 0.17	1.04 ± 0.14***	1.14 ± 0.17	
[mean‐2SD] for ALM/Ht^2^ (kg/m^2^)	7.16	6.6	7.14	6.96
95%CI of [mean‐2SD] for ALM/Ht^2^	6.9―7.4	6.4―6.8*	6.9―7.4	6.6―7.3
% of AWGS Low Muscle Mass	0.9%	6.6%†††	1.4%	4.3%
95%CI of [mean‐2SD] for ALM/BMI	0.76―0.87	0.71―0.80	0.76―0.86	0.77―0.89

BMI, body mass index; ALM, appendicular lean mass; Ht, height; CI, confidence interval; AWGS, Asian Working Group for Sarcopenia.

*** *P* < 0.001,

* *P* < 0.05, significantly lower than other values.

††† *P* < 0.001, significantly higher than other values.

**Figure 1:**
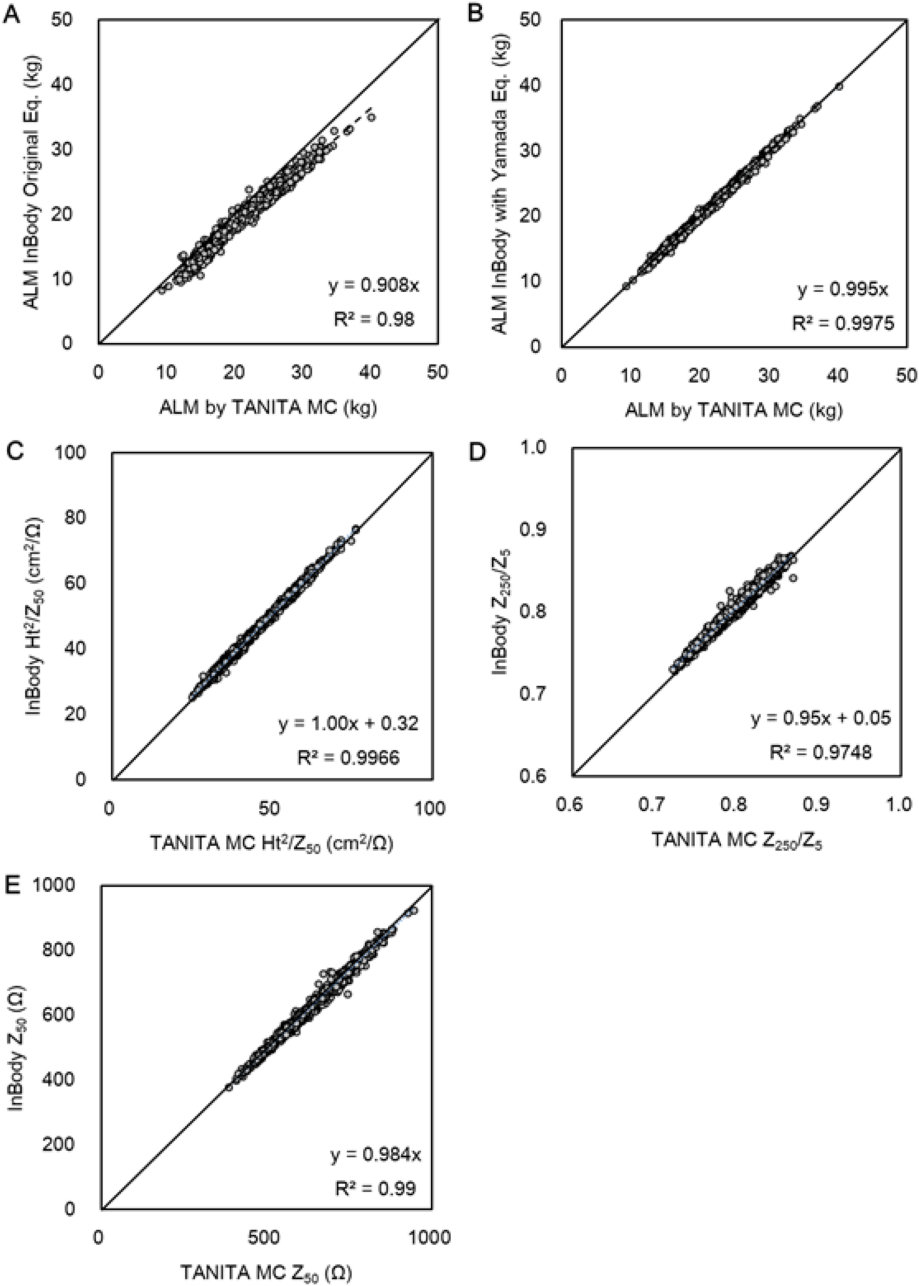
The relationship between two MF‐BIA devices. *(A)* the relationship between the ALM value estimated by TANITA MC using the equation by Yamada et al.^12^ and that estimated by InBody with its original equation is presented. The ALM value measured by InBody was significantly lower (approximately 10%) than that measured by TANITA MC (*P* < 0.001). The conversion equation to measure the ALM with TANITA MC was as follows: ALM = 1.10 x InBody. *(B)* the relationship between the ALM estimated by TANITA MC with the equation by Yamada et al. and the ALM estimated by InBody using the same equation is presented. The ALM value estimated by TANITA MC was almost identical and not significantly different from that measured by InBody. The relationship between the impedance index (height^2^/Z_50_) *(C)*, impedance ratio of low and high frequencies (Z_250_/Z_5_) *(D)*, and raw impedance (Z_50_) *(E)* of TANITA MC and InBody is presented. These values are highly correlated between the two devices. ALM, appendicular lean mass; PhA, phase angle; MF‐BIA, multi‐frequency bioelectrical impedance analysis; Z_5_, impedance at 5 kHz; Z_50_, impedance at 50 kHz; Z_250_, impedance at 250 kHz.

**Figure 2:**
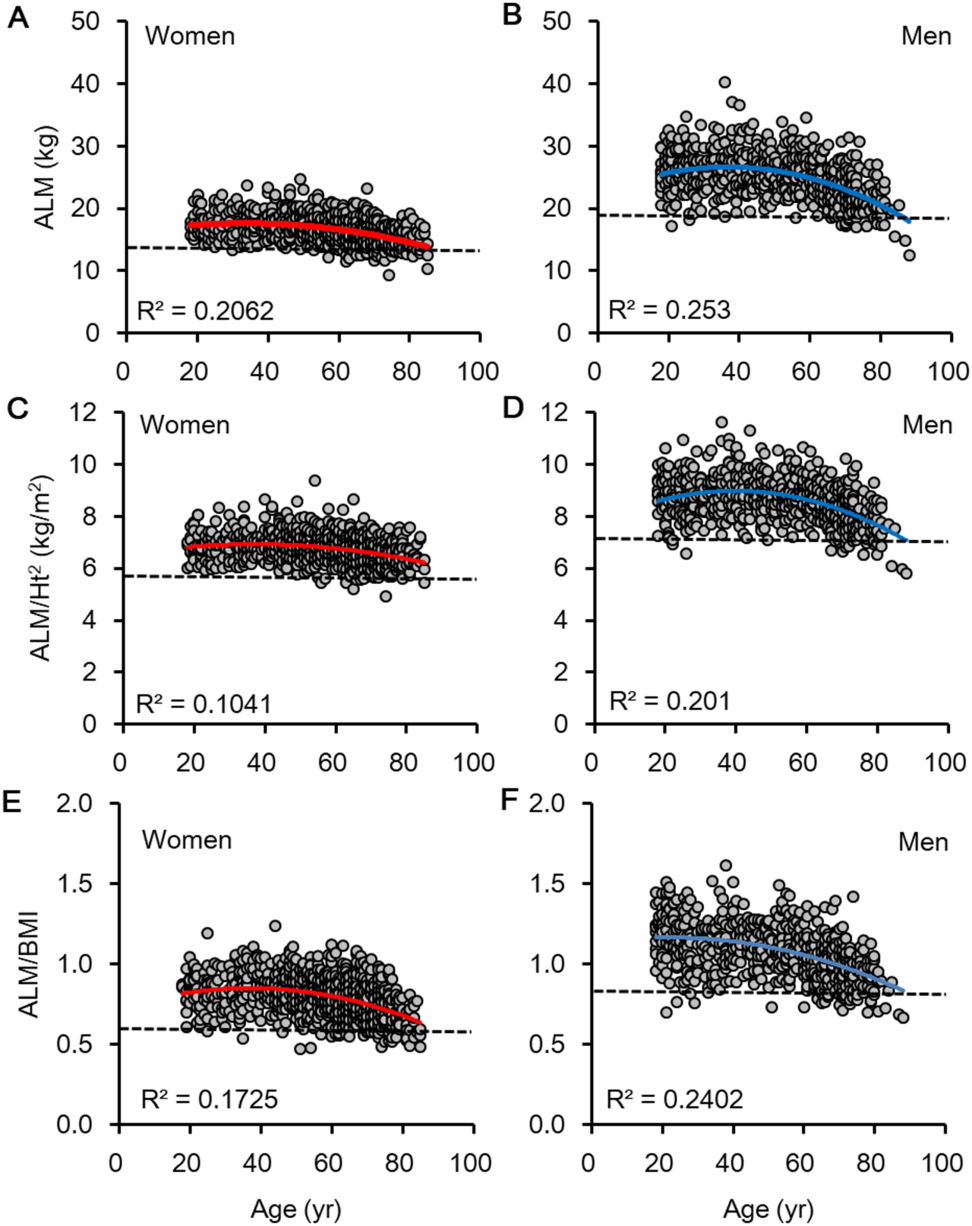
The relationship between age and the ALM, ALM/ht^2^, or ALM/BMI in women *(A, C, and E)* and men *(B, D, and F)*. Dashed line shows [mean‐2SD] of the young population. Solid curve line shows the quadratic regression line. ALM, appendicular lean mass.

Figure 1(A) shows the relationship between the ALM measured by TANITA MC and InBody with the original equation. Interestingly, the InBody ALM value was highly correlated with the TANITA MC ALM value. However, it was significantly and systematically lower (~10%) than the TANITA MC ALM value. The intercept was not significantly different from zero. However, the slope was significantly lower than 1. Thus, the cross‐calibration equation is as follows: ALM (TANITA MC) = 1.10 × ALM InBody. Figure 2(B) shows the relationship between the ALM values measured by TANITA MC and InBody recalculated using Yamada's equation.^12^ These are almost perfectly matched with each other, with few measurement errors. Figure 2(c)–(E) shows the relationship between impedance values of TANITA MC and InBody. All variables were on an identical line in the scatter plots of the two devices.

Table 3 shows the Ht, weight, BMI, ALM, ALM/Ht^2^, and prevalence of LMM in female participants aged >65 years. Weight and BMI were significantly lower in the present study than in the FNIH cohort (*P* < 0.001). The ALM/Ht^2^ values measured by TANITA MC and InBody recalculated using Yamada's equation was not significantly different from that of the ALM/Ht^2^ of the FNIH cohort (*P* > 0.05). In contrast, the ALM and ALM/Ht^2^ of InBody original (Original) values were significantly lower than those of the FNIN cohort, TANITA MC, or InBody recalculated using Yamada's equation (*P* < 0.001). The prevalence rates of LMM based on the EWGSOP2 criteria were 8.0% and 8.9% in TANITA MC and InBody recalculated using Yamada's equation, whereas that of the LMM was significantly higher in InBody (Original) (62.0%) than that in TANITA MC and InBody recalculated using Yamada's equation. When the Inbody original cutoff for LMM was applied, the prevalence of LMM was 5.6%, similar to that measured by TANITA MC and InBody recalculated using Yamada's equation. The prevalence of low ALM based on the FNIH cutoff (<15.02 kg) was very high (40.3%), whereas that of low ALM/BMI based on the FNIH cutoff (<0.512) was very low (0.7%). The prevalence of low ALM/BMI based on this study (<0.60) was 9.5%.

**Table 3:** Physical characteristics, ALM, ALM/Ht^2^, and prevalence of low muscle mass in female participants aged ≥65 years

	TANITA MC	InBody (Original)	InBody (Yamada's equation)	cf. FNIH cohort
	n = 461	*n* = 461	n = 461	n = 15,198
Age (years)	71.3 ± 4.9	71.3 ± 4.9	71.3 ± 4.9	78.6 ± 5.9
Height (cm)	153.7 ± 4.9	154 ± 4.9	154 ± 4.9	1.6 ± 0.1 (m)
Weight (kg)	50.0 ± 6.3	50.0 ± 6.3	50.0 ± 6.3	67.5 ± 13.8
BMI (kg/m^2^)	21.2 ± 2.5	21.2 ± 2.5	21.2 ± 2.5	26.9 ± 5.2
ALM (kg)	15.5 ± 1.7	13.9 ± 1.7***	15.5 ± 1.7	16.2 ± 2.9
ALM/Ht^2^ (kg/m^2^)	6.5 ± 0.4	5.9 ± 0.5***	6.5 ± 0.4	6.4 ± 1.0
ALM/BMI (m^2^)	0.74 ± 0.11	0.66 ± 0.09***	0.74 ± 0.11	―
ALM < 20th percentile	14.2	12.5	14.2	―
ALM/Ht^2^ < 20th percentile	6.19	5.40	6.19	―
ALM/BMI < 20th percentile	0.65	0.59	0.65	―
% of AWGS Low Muscle Mass (<5.7 kg/m^2^)	3.0%	38.1%†††	2.2%	―
% of EWGSOP2 Low Muscle Mass (<6.0 kg/m^2^)	8.0%	62.0%†††	8.9%	―
% of IWGS Low Muscle Mass (≤5.67 kg/m^2^)	2.2%	36.4%†††	2.2%	―
% of InBody Low Muscle Mass (<5.0 kg/m^2^)	―	3.9%	―	(*n* = 3,688)
% of FNIH/EWGSOP2 Low ALM (<15.02 kg)	40.3%	74.8%†††	41.4%	43.7%
% of FNIH Low ALM/BMI (<0.512 m^2^)	0.7%	4.1%†††	1.1%	16.7%
% of Low ALM/BMI from this study (<0.60 m^2^)	9.5%	25.6%†††	9.5%	―

BMI, body mass index; ALM, appendicular lean mass; Ht, height; AWGS, Asian Working Group for Sarcopenia; IWGS, International Working Group for Sarcopenia; EWGSOP2, European Working Group on Sarcopenia in Older People 2; FNIH, Foundation for the National Institutes of Health Sarcopenia Project

*** *P* < 0.001, significantly lower than other values.

††† *P* < 0.001, significantly higher than other values.

Table 4 shows the Ht, weight, BMI, ALM, ALM/Ht^2^, and prevalence of LMM in male participants aged ≥65 years. Weight and BMI were significantly lower in the present study than in the FNIH cohort (*P* < 0.001). The ALM/Ht^2^ values of TANITA MC and InBody recalculated using Yamada's equation were not significantly different from that of the ALM/Ht^2^ in the FNIH cohort (*P* > 0.05). In contrast, the ALM and ALM/Ht^2^ values of InBody original (Original) data were significantly lower than those in the FNIN cohort, TANITA MC, or InBody recalculated using Yamada's equation (*P* < 0.001). The prevalence rates of the LMM based on the EWGSOP2/AWGS (<7.0 kg/m^2^) were 7.1% and 7.6% in the TANITA MC and InBody cohorts, respectively. However, the prevalence of the LMM was significantly higher in InBody (Original) (19.1%) than that in TANITA MC and InBody recalculated using Yamada's equation. When the Inbody original cutoff for the LMM was applied, the prevalence of the LMM was 9.3%, similar to that observed using the TANITA MC and InBody recalculated using Yamada's equation. The prevalence of low ALM based on the FNIH cutoff (<19.75 kg) was 19.1%. However, the prevalence of low ALM/BMI based on the FNIH cutoff (<0.789 m^2^) was 7.1%. The prevalence of low ALM/BMI on this study (<0.80 m^2^) was 8.9%. We also showed the 20th percentile values of ALM, ALM/Ht^2^, and ALM/BMI in Tables 3 and 4.

**Table 4:** Physical characteristics, ALM, ALM/Ht^2^, and prevalence of low muscle mass in male participants aged ≥65 years

	TANITA MC	InBody (Original)	InBody (Yamada's equation)	cf. FNIH cohort
	n = 225	*n* = 225	n = 225	*n* = 15,198
Age (years)	71.9 ± 4.9	71.9 ± 4.9	71.9 ± 4.9	75.2 ± 6.1
Height (cm)	166.0 ± 5.4	166.0 ± 5.4	166.0 ± 5.4	1.7 ± 0.1(m)
Weight (kg)	64.0 ± 8.4	64.0 ± 8.4	64.0 ± 8.4	82.1 ± 13.5
BMI (kg/m^2^)	23.2 ± 2.5	23.2 ± 2.5	23.2 ± 2.5	27.1 ± 3.9
ALM (kg)	22.4 ± 3.0	20.8 ± 2.6***	22.2 ± 3.0	24.0 ± 3.5
ALM/Ht^2^ (kg/m^2^)	8.1 ± 0.8	7.5 ± 0.6***	8.0 ± 0.8	7.9 ± 1.0
ALM/BMI (m^2^)	0.97 ± 0.13	0.9 ± 0.11***	0.96 ± 0.13	―
ALM < 20th percentile	20.0	18.8	20.0	―
ALM/Ht^2^ < 20th percentile	7.47	7.02	7.41	―
ALM/BMI < 20th percentile	0.86	0.81	0.85	―
% of AWGS/EWGSOP2 Low Muscle Mass (<7.0 kg/m^2^)	7.1%	19.1%†††	7.6%	―
% of IWGS Low Muscle Mass (≤7.23 kg/m^2^)	11.6%	32.4%†††	13.3%	―
% of InBody Low Muscle Mass (<6.6 kg/m^2^)	―	7.1%	―	(n = 7,582)
% of FNIH/EWGSOP2 Low ALM (<19.75 kg)	19.1%	40.4%†††	20.0%	11.5%
% of FNIH Low ALM/BMI (<0.789)	7.1%	16.1%†††	8.4%	20.2%
% of Low ALM/BMI from this study (<0.82)	12.4%	24.0%†††	13.8%	―

BMI, body mass index; ALM, appendicular lean mass; Ht, height; AWGS, Asian Working Group for Sarcopenia; IWGS, International Working Group for Sarcopenia; EWGSOP2, European Working Group on Sarcopenia in Older People 2; FNIH, Foundation for the National Institutes of Health Sarcopenia Project;

*** *P* < 0.001, significantly lower than other values.

††† *P* < 0.001, significantly higher than other values.

Figure 2 shows the relationship between age and the ALM, ALM/Ht^2^, and ALM/BMI values in women and men. The dashed line shows the [mean‐2SD] of the cohort aged 18–40 years.

## Discussion

Our main aim was to validate the AWGS, EWGSOP2, FNIH, and IWGS cutoff values in a Japanese population, using one DXA and two different devices of MF‐BIA. In the DXA and TANITA MC studies, most of these cutoffs are acceptable based on the 95%CI of the [mean‐2SD] for ALM/Ht^2^ in the population aged 18–40 years. However, in the InBody, these cutoff values are significantly higher than the [mean‐2SD] of the ALM/Ht^2^ in the aforementioned population, and require different cutoff values. We obtained cutoff values of <5.0 kg/m^2^ in women and <6.6 kg/m^2^ in men for ALM/Ht^2^ of the InBody original values.

Previous studies have examined the validity of the InBody using DXA (GE Lunar or Hologic) in various populations with different age, sex, body shape, and ethnic groups.^18^, ^19^, ^20^, ^21^, ^22^ The ALM estimated by InBody was highly correlated with the ALM value obtained by DXA. However, the ALM value estimated by InBody was significantly and systematically (8–10%) lower than that obtained using DXA (Hologic QDR‐4500A) in healthy older Japanese male and female adults (*n* = 551), frail older Japanese female population (*n* = 129), and middle‐aged Caucasian population (*n* = 420).^19^, ^20^, ^21^ In contrast, the ALM estimated by TANITA MC was not significantly different from that obtained using DXA (Lunar DPX‐L, GE Healthcare).^12^ In this study, similar results were observed: the ALM estimated by InBody was significantly and systematically (8–10%) lower than that obtained using DXA (Lunar DPX‐L). However, the ALM estimated by TANITA MC was not significantly different from that obtained using DXA.

It is well known that measurement discrepancies exist between DXA devices manufactured by Horogic and Lunar. However, these discrepancies were different between the whole‐body lean mass and ALM. Shepherd et al.^23^ examined 108 adults and compared their body composition outputs using the Hologic and GE Lunar DXA systems. They found an 8% difference in the whole‐body lean mass between the Hologic (39.0 kg) and GE Lunar (36.0 kg) devices. However, they found only a 2.8% difference in the ALM between the Hologic (16.1 kg) and GE Lunar (15.7 kg) devices. The trunk lean mass was 12% different between the Hologic (22.9 kg) and GE Lunar (20.3 kg) devices. Modlesky et al.^24^ found a 2.4% difference in the whole‐body lean mass between the Hologic (57.9 kg) and GE Lunar (56.5 kg) devices. However, they also found a 4.0% difference in the ALM between the Hologic (18.4 kg) and GE Lunar (19.2 kg) devices. The trunk lean mass was 7.0% different between the Hologic (29.1 kg) and GE Lunar (27.1 kg) devices. Ito et al.^25^ found a 3.3% difference in the ALM/Ht^2^ between the Hologic (7.01 kg/m^2^) and GE Lunar (7.24 kg/m^2^) devices. Therefore, Hologic tended to show higher values in the whole‐body lean mass compared with Lunar except for ALM and ALM/Ht^2^. Moreover, discrepancies between the ALM values using the Hologic and GE Lunar devices were within 4% on average. Therefore, the current and previous results, indicating that the ALM value estimated by InBody was significantly and systematically (8–10%) lower than that obtained using DXA, cannot be explained by these differences between DXA instruments. Moreover, the current study used GE Lunar DPX‐L, while a previous study used the values obtained by Hologic QDR‐4500A, as references [19, 20]. Interestingly, both studies found that InBody provided underestimated ALM values of approximately 8–10% in Japanese population. This underestimation is nontrivial to examine sarcopenia prevalence (Tables 3 and 4). In addition, it suggests that InBody needs some corrections for examining sarcopenia. One method is to recalibrate the ALM value by InBody using a previously published equation^12^ or using a currently obtained equation as follows: recalibrated ALM = 1.10 × ALM. After recalibrating the ALM by InBody, the AWGS, EWGSOP2, and IWGS cutoff values could be used for sarcopenia definitions. In cases where we use the original ALM values obtained by InBody, the cutoff values of <5.0 kg/m^2^ in women and <6.6 kg/m^2^ in men should be used. These values are reasonable compared with those of a previous study,^17^ which showed LMM reference of <5.07 kg/m^2^ in women and <6.75 kg/m^2^ in men.

In the DXA, the 95%CI of [mean‐2SD] of ALM/Ht^2^ in the younger population was 5.2–5.8 kg/m^2^ in women and 6.6–7.3 kg/m^2^ in men. In women, the cutoff values were as follows: EWGSOP2 < 6.0, IWGS ≤5.67, and AWGS <5.4 kg/m^2^. Thus, the IWGS or AWGS has a better cutoff in the Japanese population. In men, the cutoffs were as follows: the EWGSOP2 < 7.0, IWGS ≤7.23, and AWGS <7.0 kg/m^2^. Thus, all three values are acceptable in the Japanese population. The [mean‐2SD] of ALM/Ht^2^ in the younger population was 5.49 kg/m^2^ in women and 6.96 kg/m^2^ in men, which was close to the AWGS criteria (<5.4, in women and <7.0, in men). Therefore, we suggest the use of the AWGS criteria for DXA. A previous review suggested similar values of the AWGS for the same population as DXA.^9^

In the TANITA MC, the 95%CIs of [mean‐2SD] of ALM/Ht^2^ in the younger population were 5.6–6.0 kg/m^2^ in women and 6.9–7.4 kg/m^2^ in men. In women, the cutoff values were as follows: EWGSOP2 < 6.0, IWGS ≤5.67, and AWGS <5.7 kg/m^2^. In men, the cutoff values were as follows: EWGSOP2 < 7.0, IWGS ≤7.23, and AWGS <7.0 kg/m^2^. Thus, all these three values were acceptable in men and women in the Japanese population. The [mean‐2SD] of ALM/Ht^2^ in the younger population was 5.85 kg/m^2^ in women and 7.16 kg/m^2^ in men, close to the AWGS criteria (<5.7 in women; <7.0 in men). A previous review suggested similar values of the AWGS in the same population using BIA.^9^

Here, we first showed the cutoff values for the ALM/BMI in an Asian population based on [mean‐2SD] of the younger population aged 18–40 years. BMI‐adjusted ALM is considered a variable that predicts the physical functional status better than does the absolute value of ALM. The FNIH Sarcopenia Projects used classification and regression tree analyses to establish the cutoff values.^3^ Thus, we cannot directly compare these values. However, as the mean BMI is quite different between the FNIH (27 kg/m^2^) and the Japanese or Asian cohort (Table 3), we may need to obtain different cutoff values in the Asian population. We obtained ALM/BMI cutoff values of <0.60 m^2^ in women and <0.82 m^2^ in men for DXA and TANITA MC. The cutoff value of ALM/BMI was <0.55 m^2^ in women and <0.75 m^2^ in men in InBody original values.

This study had several limitations. Especially, we only used muscle mass and did not measure muscle strength or physical performance of the participants. Therefore, we could only examine the cutoff values for LMM. Indeed, the muscle strength and physical performance are also important measurements for determining sarcopenia according to the reported guidelines.^1^, ^2^, ^7^, ^8^ The current methods are not satisfactory to obtain an accurate muscle mass measurement.^26^, ^27^, ^28^, ^29^, ^30^ Furthermore, measurement of mass per se may not distinguish between muscle wasting from chronic disease or cachexia. In addition, we only assessed a Japanese cohort, which is not representative for all Asian countries. Indeed, the body size and composition, lifestyle, and culture vary between the individuals from different Asian countries and areas. Furthermore, our study did not examine European, American, or other populations. Therefore, further studies with large sample sizes of international cohorts are needed.

Furthermore, we should note that DXA measures lean tissue mass but not skeletal muscle (cell) mass. When MF‐BIA is calibrated with DXA, the MF‐BIA also estimates LMM. Recent studies have demonstrated that DXA cannot capture age‐related decline of skeletal muscle mass very well compared with the D3‐creatine dilution method^31^, ^32^ or intracellular water measurement by bioelectrical impedance spectroscopy.^26^ These studies have suggested that DXA underestimates age‐related decline of skeletal muscle mass and the actual prevalence of sarcopenia.

Here, we also showed the 20th percentile values of ALM, ALM/Ht^2^, and ALM/BMI in healthy older adults, as reference. Regarding the whole issue of using cutoff values at the statistical extreme of a young population, the definition of sarcopenia, based on the original definition of sarcopenia proposed by Rosenberg is describing a condition of age‐related decline. However, this does not consider the impact of change in lifestyle for successive cohorts of young people (e.g., more sedentary lifestyle), such that muscle mass in younger people might not be much higher than that of older individuals. This phenomenon may explain the low prevalence of sarcopenia among a Chinese population.^33^ This is the disadvantage of placing emphasis on the measurement of muscle mass applying the same principle as that for the diagnosis of osteoporosis. The 20th percentile values of the ALM, ALM/Ht^2^, and ALM/BMI in healthy older adults may be suitable for the definition of sarcopenia. Further discussion is needed concerning this issue. Especially, validation of the cutoff values should be performed by cohort studies, which would examine the association between the SMI data and outcomes, such as fall, fracture, hospitalization, ADL dependent, and mortality.

DXA is more expensive and required maintenance and trained personnel to operate in comparison with anthropometric equations using skinfold thickness and circumference measurements.^34^ A previous study suggested that the skinfold‐circumference model had a higher accuracy than the body weight and height model in predicting total body skeletal muscle mass, but the models were population specific.^34^ Measurement of skinfold thickness and circumference of the midupper arm, midthigh, and midcalf needs exposing the limbs, but DXA and BIA do not. Particularly, BIA is inexpensive, easy to use, portable, and requires no radiation exposure. Moreover, it may be useful as a portable alternative to DXA or complex anthropometric equations using a skinfold‐circumference model.

In conclusion, this study indicated that most of the current guidelines are acceptable for DXA and TANITA MC in the Japanese population. We suggested the use of the AWGS criteria for ALM/Ht^2^ of DXA (<5.4 or <5.7 in women and <7.0 in men) and for ALM/Ht^2^ of TANITA MC (<5.7 in women <7.0 in men). ALM values measured by InBody are approximately 10% lower than those measured by DXA and TANITA MC. Here, we presented the equations that could reduce the differences obtained in calculations using the TANITA MC and InBody devices. The cutoff values of <5.0 kg/m^2^ in women and <6.6 kg/m^2^ in men should be used for ALM/Ht^2^ of InBody original values in an Asian population. We obtained an ALM/BMI cutoff value of <0.60 m^2^ in women and <0.80 m^2^ in men for DXA and TANITA MC. The ALM/BMI value was <0.55 m^2^ in women and <0.75 m^2^ in men using InBody. Because the prevalence of sarcopenia is too low particularly in women when using those criteria, the 20th percentile might be a good alternative criteria: The 20th percentile in older adult population of ALM/Ht^2^ was <6.2 and <7.5 in women and men, respectively for TANITA MC, and was <5.4 and <7.0 in women and men, respectively for InBody.

## Conflict of interest

All authors declare that they have no conflict of interest.

## Funding

This study was supported by JSPS KAKENHI with a research grant provided to Y.Y. (18H03164).

## Authors' contributions

YY, MY, and HA conceived the study; YY wrote the manuscript; MY and HA participated in data collection; YY, MY, TY, and MM analysed and interpreted the data; MY, TY, MM, and HA corrected the manuscript. All co‐authors read and approved the final version of the manuscript.

## Ethical guidelines

All authors certify that they comply with the ethical guidelines for publishing in the Journal of Cachexia, Sarcopenia and Muscle: update 2019.^35^ National and international research ethics guidelines were followed, including the Deontological Code of Ethics and the 1964 Declaration of Helsinki and its later amendments.
